# Glutathione-Induced Calcium Shifts in Chick Retinal Glial Cells

**DOI:** 10.1371/journal.pone.0153677

**Published:** 2016-04-14

**Authors:** Hercules R. Freitas, Gabriel Ferraz, Gustavo C. Ferreira, Victor T. Ribeiro-Resende, Luciana B. Chiarini, José Luiz M. do Nascimento, Karen Renata H. Matos Oliveira, Tiago de Lima Pereira, Leonardo G. B. Ferreira, Regina C. Kubrusly, Robson X. Faria, Anderson Manoel Herculano, Ricardo A. de Melo Reis

**Affiliations:** 1 Laboratory of Neurochemistry, Institute of Biophysics Carlos Chagas Filho, Federal University of Rio de Janeiro, Rio de Janeiro, RJ, Brazil; 2 Institute of Biological Sciences, Center for Health Sciences, Federal University of Rio de Janeiro, Rio de Janeiro, RJ, Brazil; 3 Institute of Medical Biochemistry Leopoldo de Meis, Federal University of Rio de Janeiro, Rio de Janeiro, RJ, Brazil; 4 Laboratory of Neurogenesis, Institute of Biophysics Carlos Chagas Filho, Federal University of Rio de Janeiro, Rio de Janeiro, RJ, Brazil; 5 Institute of Biology, Federal University of Pará, Belém, PA, Brazil; 6 Laboratory Neuropharmacology, Dept Physiology and Pharmacology, Federal Fluminense University, Niterói, Brazil; 7 Laboratory of Inflammation, Oswaldo Cruz Institute, Oswaldo Cruz Foundation (FIOCRUZ), Rio de Janeiro, Brazil; 8 Laboratory of Toxoplasmosis, Oswaldo Cruz Institute, Oswaldo Cruz Foundation (FIOCRUZ), Rio de Janeiro, Brazil; University of São Paulo, BRAZIL

## Abstract

Neuroglia interactions are essential for the nervous system and in the retina Müller cells interact with most of the neurons in a symbiotic manner. Glutathione (GSH) is a low-molecular weight compound that undertakes major antioxidant roles in neurons and glia, however, whether this compound could act as a signaling molecule in neurons and/or glia is currently unknown. Here we used embryonic avian retina to obtain mixed retinal cells or purified Müller glia cells in culture to evaluate calcium shifts induced by GSH. A dose response curve (0.1–10mM) showed that 5–10mM GSH, induced calcium shifts exclusively in glial cells (later labeled and identified as 2M6 positive cells), while neurons responded to 50mM KCl (labeled as β_III_ tubulin positive cells). BBG 100nM, a P2X7 blocker, inhibited the effects of GSH on Müller glia. However, addition of DNQX 70μM and MK-801 20μM, non-NMDA and NMDA blockers, had no effect on GSH calcium induced shift. Oxidized glutathione (GSSG) at 5mM failed to induce calcium mobilization in glia cells, indicating that the antioxidant and/or structural features of GSH are essential to promote elevations in cytoplasmic calcium levels. Indeed, a short GSH pulse (60s) protects Müller glia from oxidative damage after 30 min of incubation with 0.1% H_2_O_2_. Finally, GSH induced GABA release from chick embryonic retina, mixed neuron-glia or from Müller cell cultures, which were inhibited by BBG or in the absence of sodium. GSH also induced propidium iodide uptake in Müller cells in culture in a P2X7 receptor dependent manner. Our data suggest that GSH, in addition to antioxidant effects, could act signaling calcium shifts at the millimolar range particularly in Müller glia, and could regulate the release of GABA, with additional protective effects on retinal neuron-glial circuit.

## Introduction

Retinal tissue is devoted to transduction of light stimulus in visual information and it is constituted by six different kinds of neurons and one major glia in a complex network. Retinal Müller cells are the major glia component and an active compartment that interacts with most, if not all, neurons in the vertebrate retina [1]. Neuronal-glial interactions are critical to the retina physiology, and they are mediated by contact at different layers or by different agents, such as neuro- and gliotransmitters, trophic factors, or agents such as glutathione (GSH), one of the most abundant low-molecular-weight antioxidant in the retinal tissue. Lack of proper control of redox homeostasis can be implicated in the etiology and/or progression of a wide range of human diseases, including cancer, aging, and neurodegenerative diseases [2]. In addition, it was previously described that retinal injuries such as diabetic retinopathy, glaucoma or macular edema have been linked to disturbances in antioxidant defenses [3].

Compartmentalization of brain GSH between neurons and glia has been a matter of debate in the past, and evidence indicate that this agent is found at millimolar levels as an important protection against many reactive oxygen species (ROS). While ascorbic acid (AA) is an enzymatic co-factor and antioxidant predominantly expressed in neurons, GSH is a general molecule present in the brain cells. Studies performed in hippocampus, cerebellum and brain cortex describe that GSH levels reaches up to 4mM in glial cells while in neurons these values do not exceed 2.5mM [4], [5]. AA and GSH uptake from synaptic cleft is mediated by sodium vitamin C co-transporter 2 (SVCT-2) [6] and GLAST transporter respectively. SCVT-2 is expressed in neurons while GLAST is largely expressed in Müller glia [7]. The activity of both transporters is well characterized as Na^+^-dependent.

Despite their similar antioxidant properties, the differential distribution between neurons and glia suggests that GSH and AA might have complementary but distinct roles in the CNS. Supporting this hypothesis, it is well documented that both GSH and AA activate different signaling pathways in neuro-glial circuits [5]. Indeed, it has been postulated that GSH may serve additionally as a neuromodulator/neurotransmitter in the brain, reviewed in [2, 8, 9]. Evidence of high-affinity binding sites for GSH and GSH derivatives are described in pig cerebral cortical synaptic membranes [10]. Moreover, thiol-containing compounds may participate in reduction–oxidation (redox) reactions altering biophysical properties of various ionotropic receptors and ion channels. Indeed, several findings support the hypothesis that GSH and its derivatives have some affinity for the NMDA recognition domain in a manner independent of the thiol moiety (reviewed in [9]. It was also shown that GSH releases [^3^H] dopamine in mouse striatal slices [8]. In addition, astrocytes have the ability to secrete GSH into the extracellular space [11], [12]. GSH produced by glial cells might be transported to neurons [13] as specific neuronal transporters to uptake GSH have been described [14].

It has been shown that aspartate releases GSH through reversal of the GLAST transporter in the chick retina [7]. This could be a potential protective mechanism on the retina during excitotoxicity. Now we show that GSH (at the millimolar range) release GABA from chick retina. Also, 5mM GSH stimulated Ca^2+^ variations only in the glial compartment, in a P2X7 but not ionotropic (NMDA and non-NMDA) glutamate receptor dependent manner. Also, mixed retinal cells or purified Müller cells in culture released [^3^H] GABA when stimulated with GSH in a P2X7 dependent manner. Moreover, propidium iodide (PI) uptake by Müller glia induced by GSH or ATP was inhibited by P2X7 receptor antagonists or by the calcium cell permeant chelator, BAPTA-AM. These results could provide an important issue in neuroprotection or as a redox loop in the neuronal glia circuit.

## Material and Methods

All experiments involving animals were approved by and carried out in accordance with the guidelines of the Institutional Animal Care and Use Committee of the Federal University of Rio de Janeiro (permit number IBCCF-035), and all efforts were made to minimize suffering. Fertilized White Leghorn chicken eggs were obtained from a local hatchery. In this study, we used around 55 eggs (110 retinae). Embryos were staged according to [15] and sacrificed by decapitation on embryonic day 8 (E8). The eyes were removed and the retinas dissected out in a Ca^2+^- and Mg^2+^-free Hanks' (CMF) solution. We confirm that all animal use and experimental procedures were carried out in accordance with the guidelines of the Brazilian Society of Neuroscience and Behavior (SBNeC). Dulbecco's modified Eagle's medium (DMEM), fetal calf serum (FCS) and gentamycin were obtained from Gibco (USA). MK801 (Cat. No.0924) and DNQX were obtained from Tocris-Cookson (St. Louis, MO USA), ATP, propidium iodide, GABA, A-740003 (N-(1-{[(cyanoimino)(5-quinolinylamino) methyl] amino}-2,2-dimethylpropyl)-2-(3,4-dimethoxyphenyl)acetamide, Brilliant Blue G (BBG), thapsigargin, glutathione were from sigma. BAPTA-AM, CM-H2DCFDA and Fura-2 AM were obtained from Molecular Probes. [^3^H] GABA was from Amersham Pharmacia Biotech, Piscataway, NJ, USA. All other reagents were of analytical grade.

### Mixed Retinal cells in culture

Primary low-density cell cultures of retinal cells (10^6^ cells/35 mm dish) at stage E8C8 (8 day embryo and 8 days *in vitro*) were prepared as described previously [16, 17]. Briefly, 8-day old chick embryo (E8) retinas were dissected, cleared of pigmented epithelium and placed on a Ca^2+^- and Mg^2+^-free salt balanced medium (CMF). Trypsin (final concentration of 0.05% w/v) was added to the medium and incubated at 37°C for 10 min. After brief centrifugation, the pellet was suspended in 5 ml of DMEM + F12 medium plus 5% fetal calf serum (FCS) and mechanically dissociated by pipetting the tissue. Cells were plated on 15mm coverslips (Marienbad, German) previously treated with Poly-L-Lysine (to obtain neuronal enriched cultures, at 1% FCS). The plates were then transferred to a humidified atmosphere of 95% air/5% CO_2_ in an incubator. The medium was changed once 24h after the cells were plated and then at every 72h. Alternatively, retinal cells in high density (> 5 x 10^6^ cells) were plated on coverslips with DMEM + F12 medium plus 10% FCS to obtain mixed retinal cells in culture.

### Retinal Müller Glia

Retinal Müller glial cultures were generated from E8 or E9 chick retina essentially as described before [18]. Briefly, chick retina was dissociated using trypsin, and cells were seeded (density of 4 x 10^5^ cells/cm^2^) over 6 well dishes 35 mm culture dishes in DMEM containing 10% FCS. After 10 days, cell cultures were virtually free of neurons. Cultures were subsequently washed with DMEM, and purified glial cultures were obtained after approximately 12 days.

### Single cell calcium imaging

Variations of free intracellular calcium levels ([Ca^2+^]i) were evaluated in single cells obtained from retinal cells in culture adapting the protocol from [19], essentially described in [20]. Retinal cells in culture were loaded for 40 min with 5μM Fura-2/AM (Molecular Probes), 0.1% fatty acid-free bovine serum albumin (BSA), and 0.02% pluronic acid F-127 (Molecular Probes) in Krebs solution (132mM NaCl, 4mM KCl, 1.4mM MgCl_2_, 2.5mM CaCl_2_, 6mM glucose, 10mM HEPES, pH 7.4), in an incubator with 5% CO_2_ and 95% atmospheric air at 37°C. After a 10-min post-loading period at room temperature in Krebs solution (KS), to obtain a complete hydrolysis of the probe, 15 mm coverslip (Marienbad, Germany) with the cells was mounted on a chamber in a PH3 platform (Warner Instruments, Hamden, CT) on the stage of an inverted fluorescence microscope (Axiovert 200; Carl Zeiss). Cells were continuously perfused with KS and stimulated with different solutions (GSH or any other). Solutions were added to the cells by a fast-transition system (4 sec). The variations in [Ca^2+^]i were evaluated by quantifying the ratio of the fluorescence emitted at 510 nm following alternate excitation (750 milliseconds) at 340 and 380nm, using a Lambda DG4 apparatus (Sutter Instrument, Novato, CA) and a 510 nm long-pass filter (Carl Zeiss) before fluorescence acquisition with a 40X objective and a Cool SNAP digital camera (Roper Scientific, Trenton, NJ). Acquired values were processed using the MetaFluor software (Universal Imaging Corp., West Chester, PA). Values for Fura-2 fluorescence ratio were calculated based on a cut-off of 10% increase in the [Ca^2+^]i level induced by the stimulus. Cell cultures after single cell imaging were fixed in 4% paraformaldehyde (PFA).

### Immunocytochemical Staining for Phenotype Discrimination

Retinal cells in culture were fixed in 4% PFA, for 30 min at room temperature, and then washed twice with phosphate buffer saline (PBS), for 5 min. Cultures were permeabilized, and nonspecific binding sites were blocked with 0.25% Triton X-100 and 3% BSA, dissolved in PBS for 30 min at room temperature. Retinal cells were subsequently incubated overnight at 4°C with the following primary antibodies, all of which prepared in PBS containing 0.1% Triton X-100 and 0.3% BSA: rabbit anti-Tuj-1 (1:200; Abcam), mouse monoclonal anti-2M6 (1:300), Thereafter, the coverslips were rinsed in PBS and incubated for 2 h at room temperature with the appropriate secondary antibodies (all from Molecular Probes). After an additional rinse in PBS, retinal cell nuclei were stained with DAPI (1 μg/ml in PBS containing 0.25% BSA), for 2 min at room temperature. Finally, the preparations were mounted using Dako-Cytomation fluorescent medium.

### HPLC analysis of GABA release

Chick retinal tissues were incubated in Hank´s solution for 15 minutes at 37°C in the presence of GSH (1mM or 5mM) or buthionine sulfoximine (BSO) at 50μM. After that, extracellular GABA levels were measured using a HPLC instrument (model LC20-AT; Shimadzu, Japan) following precolumn derivatization with o-phthalaldehyde and separation on a C18 reverse-phase chromatographic column (Shimadzu, Shim-Pack VP-ODS, 250 x 4.6 mm, Japan) coupled to a fluorescence detector (model RF-10AXL) with excitation wavelength 340 nm and emission wavelength 460 nm. Buffers and the gradient system were as follows: the mobile phase was solvent A (0.8 M sodium acetate in 10% methanol) and solvent B (70% methanol in 30% water) at 1 mL/min flow rate. The mobile phase was maintained at 100% A for 1 min from the initiation of the program; 70% A/30% B in 10 min; 50% A/50% B in 20 min; 100% A in 25 min. Samples were further mixed with 20 μL of 1% trichloroacetic acid. After centrifugation (5000 rpm for 10 min), 20 μL of homoserine (internal standard) were added to 300 μL of the supernatant. Samples were derivatized (60 μL of sample; 40 μL of 0,1 M sodium borate pH 9.5 and 10 μL of o-phtalaldehyde) and 5 min later 20 μL of this mixture was injected into an HPLC column at room temperature. GABA quantification was based on peak high by comparison with an external calibration curve prepared from solutions of known GABA concentrations.

### [^3^H] GABA release

The protocol was essentially performed as previously described [21]. Briefly, retinas were incubated with 1mL DMEM containing 0.1μCi [^3^H]-GABA for 60 min at 37°C. Non-incorporated radioactive GABA was removed by successive washings with modified Hank’s solution. Afterwards, mixed neuron-glia or Müller glia cells in culture were transferred to 1 mL cups and superfused with medium at a rate of 0.5 mL/10min at 37°C. The basal superfusion medium contained the modified Hank’s solution and, when specified, drugs of study. The stimulated superfusion medium was similar to the basal one with the exception of GSH or L-aspartate added as a stimulus for GABA release. After the superfusion, the radioactivity released in the medium was quantified by liquid scintillation and the remaining radioactivity in the cells was counted after cell disruption with distilled water followed by successive freeze-thaw cycles. The amount of [^3^H]-GABA released was plotted as the percentage of the total radioactivity taken up by the cells.

### Dye Uptake by Müller cells in culture

Avian Müller cells permeabilization was visualized and quantified by the differential uptake of 500 ng/mL propidium iodide (PI) essentially as described before for cultured mice astrocytes [22]. Müller glia cells were plated in a 96-well plate (Corning, SP, Brazil) and incubated at 37°C with ATP 1mM, GSH 5mM or GSH in the presence of P2X7 receptor antagonists or BAPTA-AM, which were added 10 min before. Then, cells were incubated with GSH for 20 min. The fluorescent dye was added during the last 5 min of ATP or GSH incubation. After these procedures, the cells were washed, the medium was replaced with extracellular saline solution, and the cells were imaged by fluorescence microscopy (Nikon, Eclipse TS2000, Tokyo, Japan). Data were analyzed with Image J software, Version 4.02 (National Institutes of Health). The positive cells displayed gray color under the same conditions used for the negative control (untreated cells). Experiments were performed in triplicate.

### Measurement of GSH concentrations

For the assessment of GSH concentrations, Müller’s glia cells were incubated in the absence or presence of 5mM GSH for 60 seconds. Then, cells disposed in a 35 mm well were washed three times with KS and homogenized in 500μL of 20mM PBS, pH 7.4, containing 140mM KCl. GSH concentrations were measured according to [23], with slight modifications [24]. Calibration curve was prepared with standard GSH (0.01–1mM, Sigma) and the concentrations were calculated as nmol/mg of protein.

### Live and Dead assay

A fluorescence-based viability assay was performed exactly as [20] (Live-dead assay; Invitrogen, Carlsbad, CA).

### Detection of DNA fragmentation by TUNEL technique

Fixed cells were incubated in 0.5% Triton X-100 in PBS for 15 min and then rinsed with PBS, pH7.4, for 10 min. After that, cells were pre-incubated with terminal-deoxyribonucleotidyl-transferase (TdT) Buffer for 20 min at room temperature, followed by incubation in a solution of TdT Enzyme in Reaction Buffer from the Kit S7111-The ApopTag^®^ Plus Fluorescein *in situ* Apoptosis Detection Kit (Millipore) according to manufacturer's directions. After 2 hours, at 37°C, at humid atmosphere, the reaction was terminated by incubation with Stop/Wash Buffer for 10 min at room temperature. Thereafter, cells were rinsed with PBS for 5 min and incubated with anti-Digoxigenin-FITC in blocking Solution for 1 hour, at room temperature, protected from the light. Cells were washed with PBS for 10 minutes. For nuclear staining, cells were incubated with DNA-intercalating dyes 4'-6-di-amino-2-phenylindole (DAPI) solution for 1 minute and then washed with PBS for 5 min. Coverslips was mounted with glycerol N-propylgallate. For positive control, cells were treated with the inhibitor of the endoplasmic reticulum calcium ATPase thapsigargin. For negative control, cells received similar treatment, but TdT enzyme was omitted.

### CM-H2DCFDA oxidation assay

Intracellular ROS accumulation was evaluated in live cells using a chloromethyl derivative of H2DCFDA, CM-H2DCFDA (Molecular Probes) useful as an indicator for reactive oxygen species (ROS) in cells, as previously reported [25]. Müller glial cells were plated on six-well plates and then incubated in the absence or presence of GSH 5mM for 60 seconds. Cells were washed three times with KS and then exposed to 0.1% H_2_O_2_ for 30 minutes. Cells were then incubated with 5μM CM-H2DCFDA in PBS for 30 min at 37°C in the dark, washed three times with KS and examined under a fluorescence microscope. Analysis of fluorescence was carried out using Image J. At least five independent experiments were performed for each condition.

### Data analysis

Statistical analysis was performed (Prism 6) using Student's t test or analysis of variance (ANOVA) followed by post hoc Tukey´s test for multiple comparisons, or Dunnett's multiple comparison post-hoc tests. Statistical significance was taken at p <0.05. Unless specified otherwise, the results are expressed as mean ± standard error (SEM) of at least three separate experiments performed in triplicate.

## Results

### Intracellular calcium responses to GSH and KCl

**Fig 1:**
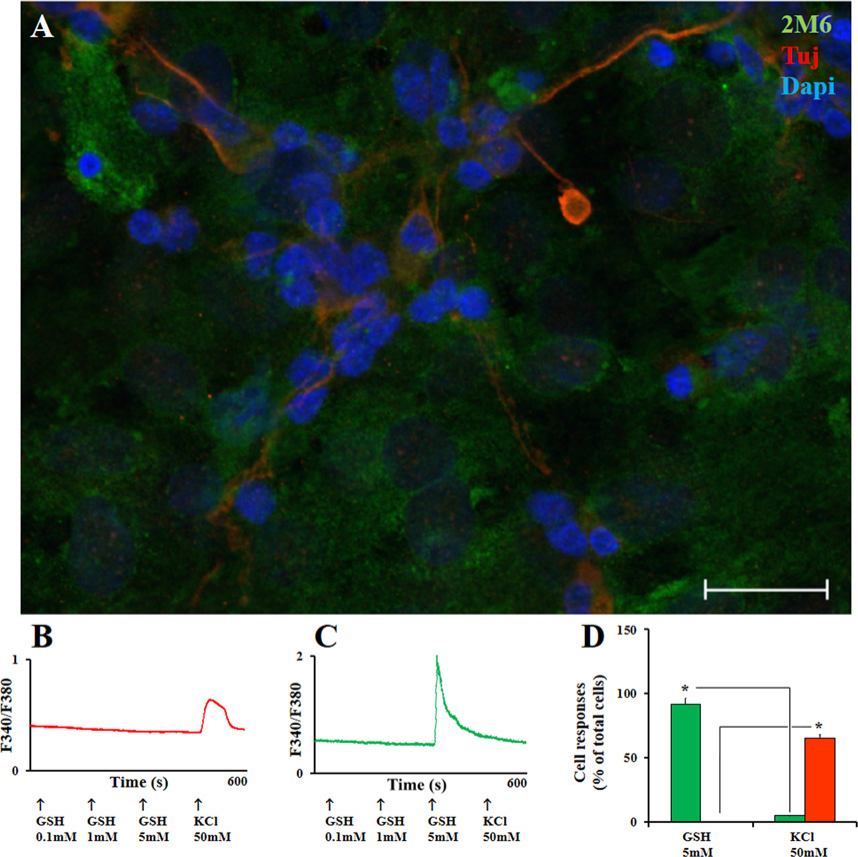
Immunocytochemistry of avian mixed neuron-Müller glial cells in culture and calcium responses by selective agents. Mixed neuron-glia retina cells in culture. A. Neurons were immunostained with the Tuj-1 antibody (β_III_ Tubulin, red) while glia cells were labeled with 2M6 (green). Nuclei were labeled with DAPI (blue). B. Cell calcium imaging traces of emission fluorescence (ratio 340/380 nm excitation) of a representative β_III_ tubulin labeled neuron (red) activated by 50mM KCl. C. A representative 2M6 labeled glial cell (green) activated by 5mM GSH; D. Quantification of the functional responses of 2M6+ cells (glia) show that 92% were responsive to 5mM GSH and none responded to 50 mM KCl; Alternatively, 64% of Tuj-1 β_III_ Tubulin+ cells were activated by 50mM KCl; at least 280 cells were analyzed (n = 5). A. 50μm scale bar. *Calculated significance was at least p < 0.05.

GSH is one of the main antioxidants in the CNS. In order to investigate if GSH signals to neurons and glial cells in terms of calcium shifts, we performed single cell calcium imaging (SCCI) to obtain variations of free intracellular calcium concentrations using fura2-AM as a probe. As shown (Fig 1), mixed neuronal and Müller glial cells responses to GSH and KCl were quite different. While 50mM KCl induced a selective activation increasing intracellular Ca^2+^ levels only in neuronal cells, the great majority of Müller glia cells were responsive to 5mM GSH (but not under 1mM). In order to validate the functional responses of neuronal-glial cells in culture, we performed immunocytochemistry of the same registered cells. As shown (Fig 1B), all the KCl activated cells were labeled to anti-β_III_ tubulin (a neuronal marker) while the cells that responded to GSH were labeled to anti-2M6, an antibody that recognizes avian Müller glia cells [26]. Fig 1 shows the quantification for the responses for glial and neuronal cells in terms of calcium variations induced by GSH or KCl.

### Müller cells are activated by GSH

We have developed different types of retinal cultures to study neuronal-glial properties: (a) Mixed neuron–glial cells (prepared in high density, with ~20 × 10^6^ cells), (b) enriched neuronal cells prepared in low density, with ~2 × 10^6^ cells or less, seeded on treated Poly-L-lysine (10 μg/mL) plastic dishes and (c) purified Müller glia culture (5×10^6^ cells) in DMEM containing 10% FCS and cultured for 10 days, when neurons are eliminated [17].

**Fig 2:**
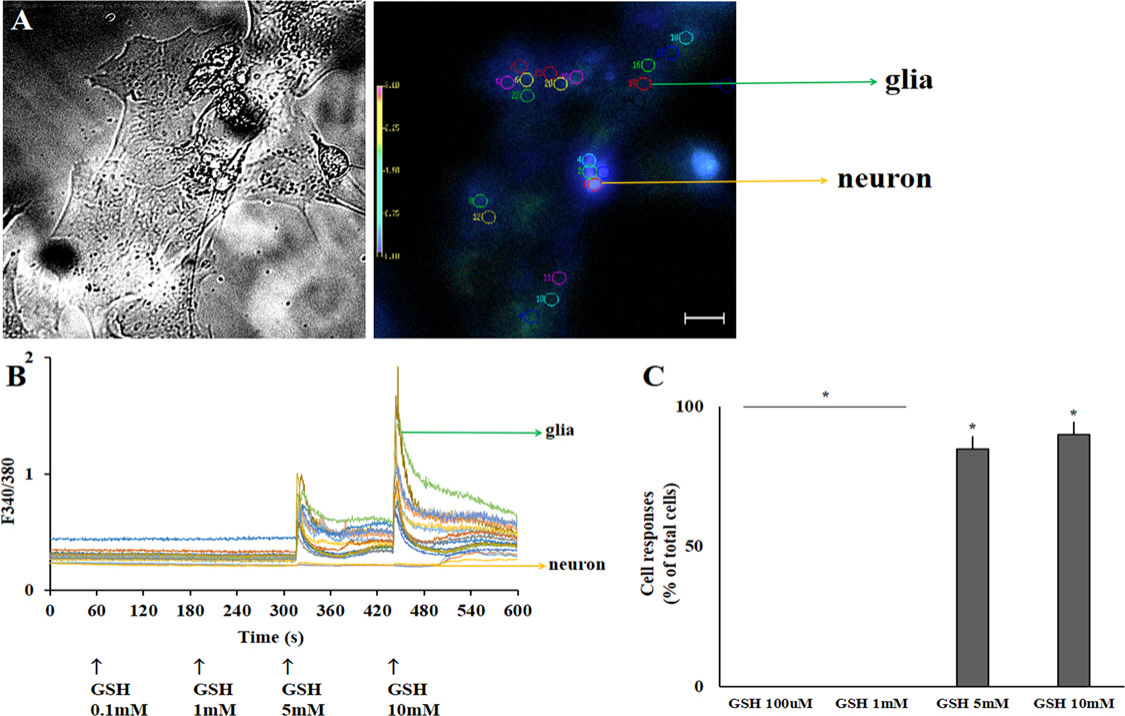
GSH-induced calcium shifts in enriched glial retinal culture cells. A. Mixed neuron-glia cells in culture in bright field or in fura-2 loaded fluorescence. Neurons are identified by brighter soma compared to glia. B. Cells were chosen (colored circles) and a dose response curve for GSH-induced calcium responses is seen for each cell. As shown, only glia cells respond to GSH, indicated in the arrows below (5–10mM). C. Panel C shows that the majority of glial cells respond to GSH. At least 350 cells were analyzed (n = 6). A. 50μm scale bar. *Calculated significance was at least *p* < 0.05.

**Fig 3:**
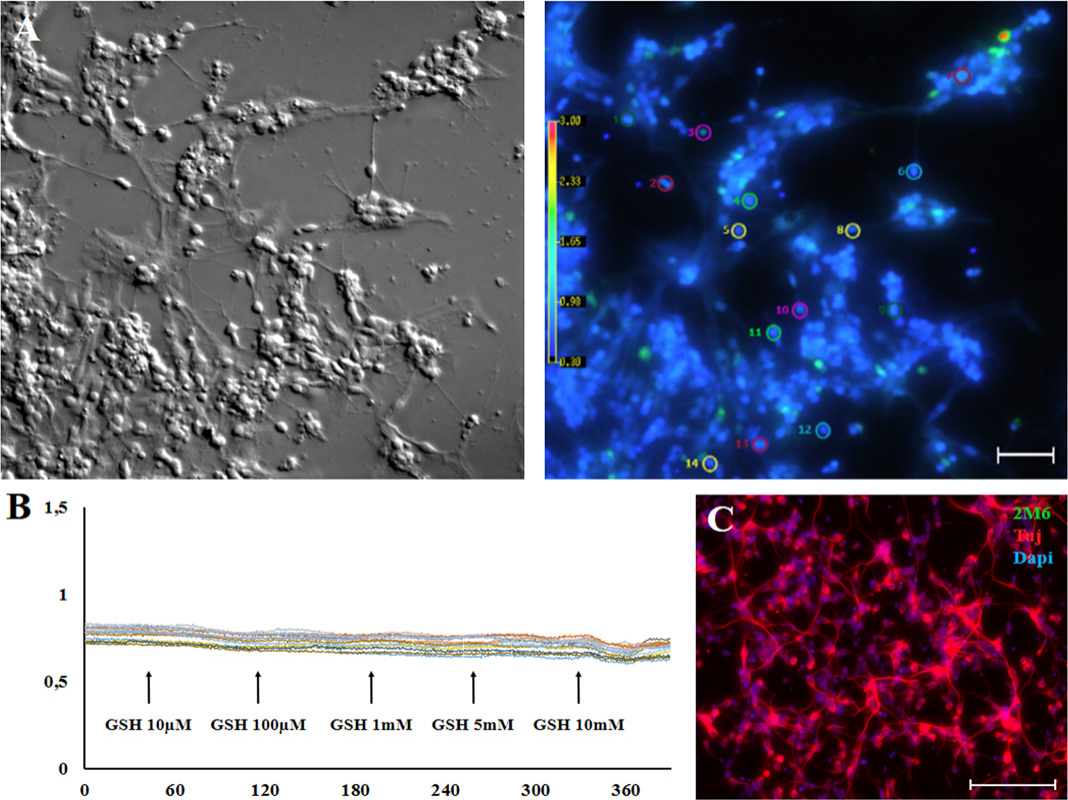
Lack of response of GSH in enriched neuronal retinal culture cells. A. Neuron-enriched retinal cultures in bright field or in fura-2 loaded fluorescence. B. Identified neuronal cells had no Ca^2+^ response when activated with GSH (10μM-10mM), 100μm scale bar. Total of 394 cells were evaluated (n = 3). C. Neurons were immunostained with the Tuj-1 antibody (β_III_ Tubulin, red) while glia cells were labeled with 2M6 (green). DAPI was used to label nuclei (blue).

Dose-response curve (from micro to millimolar) to GSH were elaborated in enriched glial retinal culture cells. As shown in Fig 2, only glial cells were activated by GSH at the millimolar range (5 to 10mM). Alternatively, we tested if GSH could activate enriched neuronal retinal cultures (Fig 3A). As shown, GSH (10μM-10mM) failed to induce Ca^2+^ shifts when neuronal cells were evaluated (Fig 3B). Neuronal cells were immunostained with the Tuj-1 antibody (β_III_ Tubulin, red) while DAPI was used to label nuclei (blue) (Fig 3C). Therefore, our data suggest that GSH selectively activates Müller glia cells in retinal culture.

### P2X7 purinergic receptors are involved in the GSH response in glial cells

Avian retinal mixed neuron and Müller glia cells in culture express both glutamatergic [20, 27] and purinergic [28] receptors that are permeable to calcium ions. Therefore, to evaluate the involvement of these receptors on the calcium-induced response of antioxidants, antagonists for ionotropic glutamate receptors MK-801 (NMDA) and DNQX (non-NMDA) or for P2X7 purinergic receptors, Brilliant blue G (BBG), were incubated with 5mM GSH in purified Müller glia culture cells.

**Fig 4:**
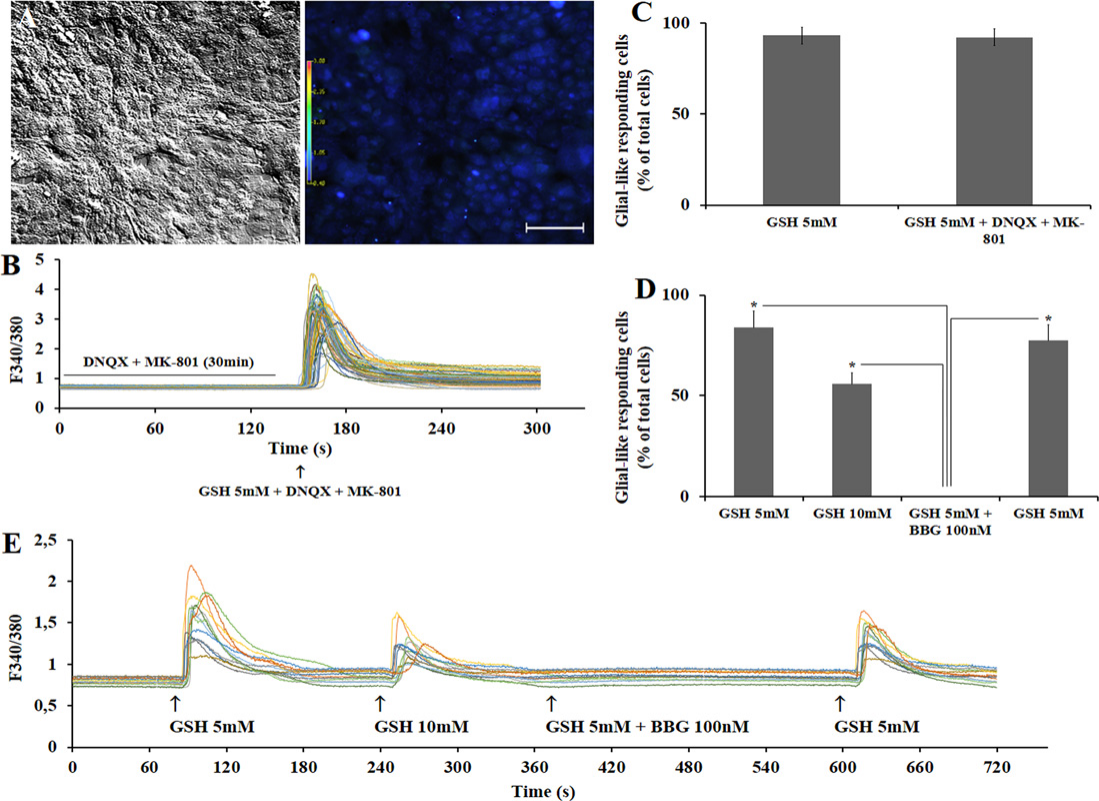
Effect of ionotropic NMDA (MK-801) and non-NMDA (DNQX) or P2X7 (BBG) receptor antagonists on calcium responses induced by GSH in glial cells. A. Cultured Müller cells in bright field or in fura-2 loaded fluorescence. B. A 30 minutes pre-incubation with 70μM DNQX + 25μM MK-801 followed by a single GSH (5mM) stimulus with or without antagonists. C. After quantification, both GSH (5mM) stimuli were able to evoke calcium responses in Müller glial cells. Total of 117 cells were analyzed for GSH 5mM without inhibitors (n = 3), while 115 cells were evaluated for GSH 5mM in the presence of inhibitors (n = 3). D. Data quantification of calcium responses shows that no cells are activated by 5mM GSH in the presence of 100 nM BBG. E. Calcium transient is recovered when 100nM BBG is washed and only 5mM GSH is incubated. No statistical differences were found between samples in C. *Calculated significance was at least *p* < 0.05. A. 100μm scale bar.

As shown in Fig 4, Ca^2+^ transient stimulus is observed when Müller glia is activated with GSH 5mM in the absence or presence of 25 μM MK-801 + 70μM DNQX (20 min incubation). On the other hand, the purinergic receptor antagonist 0.1μM BBG blocked the ability of millimolar concentrations of GSH to raise intracellular calcium when added 20 minutes before GSH. Incubation of BBG completely blocks the calcium shift induced by 5mM GSH or by 1mM ATP.

### Effect of oxidized glutathione on calcium transients in Müller glial cells

**Fig 5:**
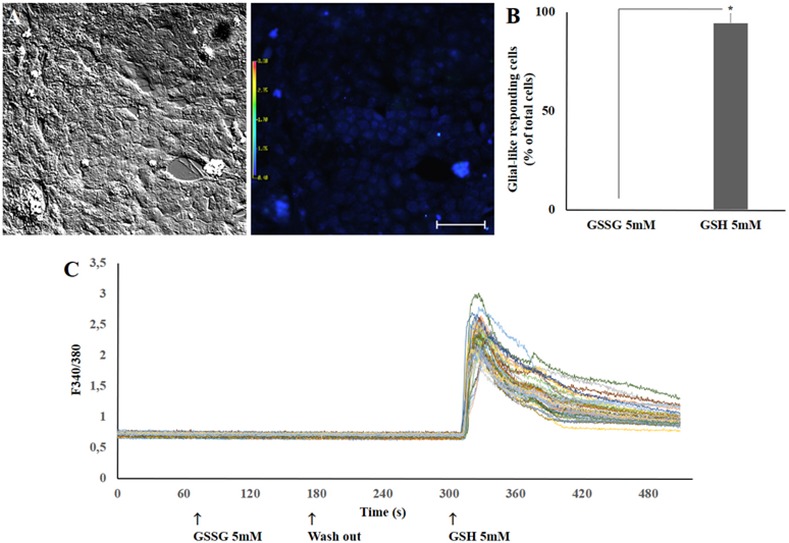
Oxidized glutathione (GSSG) and GSH effects on intracellular calcium transients in glial cells at millimolar concentrations. A. Cultured Müller cells in bright field or in fura-2 loaded fluorescence. B. Dose-response curve for 5mM GSSG and 5mM GSH incubation. C. Quantification data shows that 5mM GSSG is unable to evoke calcium transients in glial cells while GSH stimulates virtually all imaged single cells in the field. Total of 128 cells analyzed (n = 3). *Calculated significance was at least *p* < 0.05. A. 100μm scale bar.

We asked if oxidized glutathione (GSSG) could also activate Ca^2+^ shifts in Müller cells in culture. Fig 5 presents a comparison between the effect of 5mM oxidized glutathione (GSSG) and 5mM GSH in stimulating calcium transients in Müller cells. As shown, GSSG failed to induce calcium transients, while GSH greatly promoted this effect, indicating that the antioxidant and/or structural features of GSH are essential to promote elevations in cytoplasmic calcium levels.

### Extracellular GABA levels are regulated by GSH treatment

**Fig 6:**
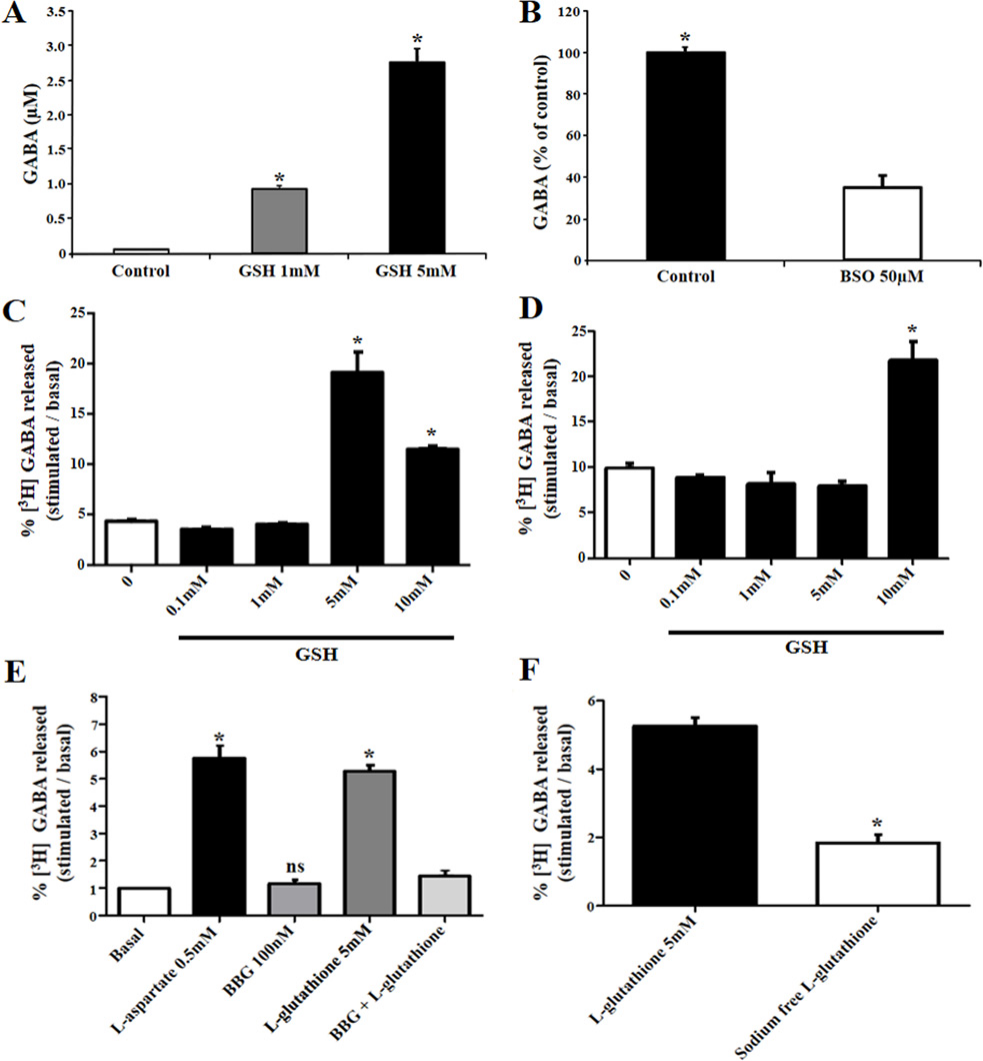
GSH evokes increase in the extracellular GABA levels in chick retinal tissue and in mixed neuronal-glial cells in culture. GSH treatment evokes GABA release in chick retinal tissue or in cells in culture (A) Extracellular GABA levels were measured in E11 avian retina upon incubation with 1–5mM GSH for 10 min. Values are means ± SD from two independent experiments (ANOVA-Tukey *p < 0.01 vs. control, n ≥ 4). **(**B) Extracellular GABA is decreased in retinal tissue treated with inhibitor of GSH synthesis (25μM BSO) for 24h promote an intense decrease in the GABA release from retinal cells. Values are means ± SD expressed as percentage of control from two independent experiments (ANOVA-Tukey *p < 0.01 vs. control, n ≥ 4). (C) Percentage of [^3^H] GABA release in the presence of increasing concentrations of GSH in mixed neuronal-glial cells or in (D) purified Müller cells in culture. (E) Percentage of [^3^H] GABA release induced by 5mM GSH in mixed neuronal-glial cells is blocked by 100nM BBG. [^3^H] GABA was also released by L-aspartate as a positive control. *significance was *p*<0,05. Total of three individual experiments (n = 3). (F) Percentage of [^3^H] GABA release in the presence of 5mM GSH in Hanks solution or in Na^+^-free Hanks solution Values are means ± SEM expressed from three independent experiments (ANOVA-Tukey *p < 0.01 vs. control, n ≥ 4).

GABA is the major inhibitory neurotransmitter in the retinal circuit. We asked if GSH could release GABA in the retinal tissue. Our results demonstrated that GSH evokes a concentration dependent increase in the extracellular levels of GABA in embryonic retinal tissue (Fig 6A). Added to this, we also observed that treatment with an inhibitor of GSH synthesis, BSO at 50μM, decreases significantly the extracellular concentration of GABA (Fig 6B). Since GSH induced Ca^2+^ shifts selectively in Müller cells, we asked if [^3^H] GABA could be released in purified Müller glia or in mixed neuronal-glial cells in culture. As shown, GSH induced [^3^H] GABA release in a P2X7 dependent manner in mixed neuron-glia cells (Fig 6C) or in purified Müller cells in culture (Fig 6D). Also, 100 nM BBG completely blocked this response induced by GSH (Fig 6E). In addition, this response was also inhibited when sodium ions were substituted with Tris (Fig 6F). Therefore, our data suggest that GABA is released by GSH in retinal cells, and the source of GABA is more likely to be of glial origin in the chick retina. However, we cannot discard the release of GABA from neurons, as the mixed retinal culture was also effective in terms of release.

### GSH induces Müller glia permeabilization in a P2X7 receptor and intracellular calcium dependent manner

**Fig 7:**
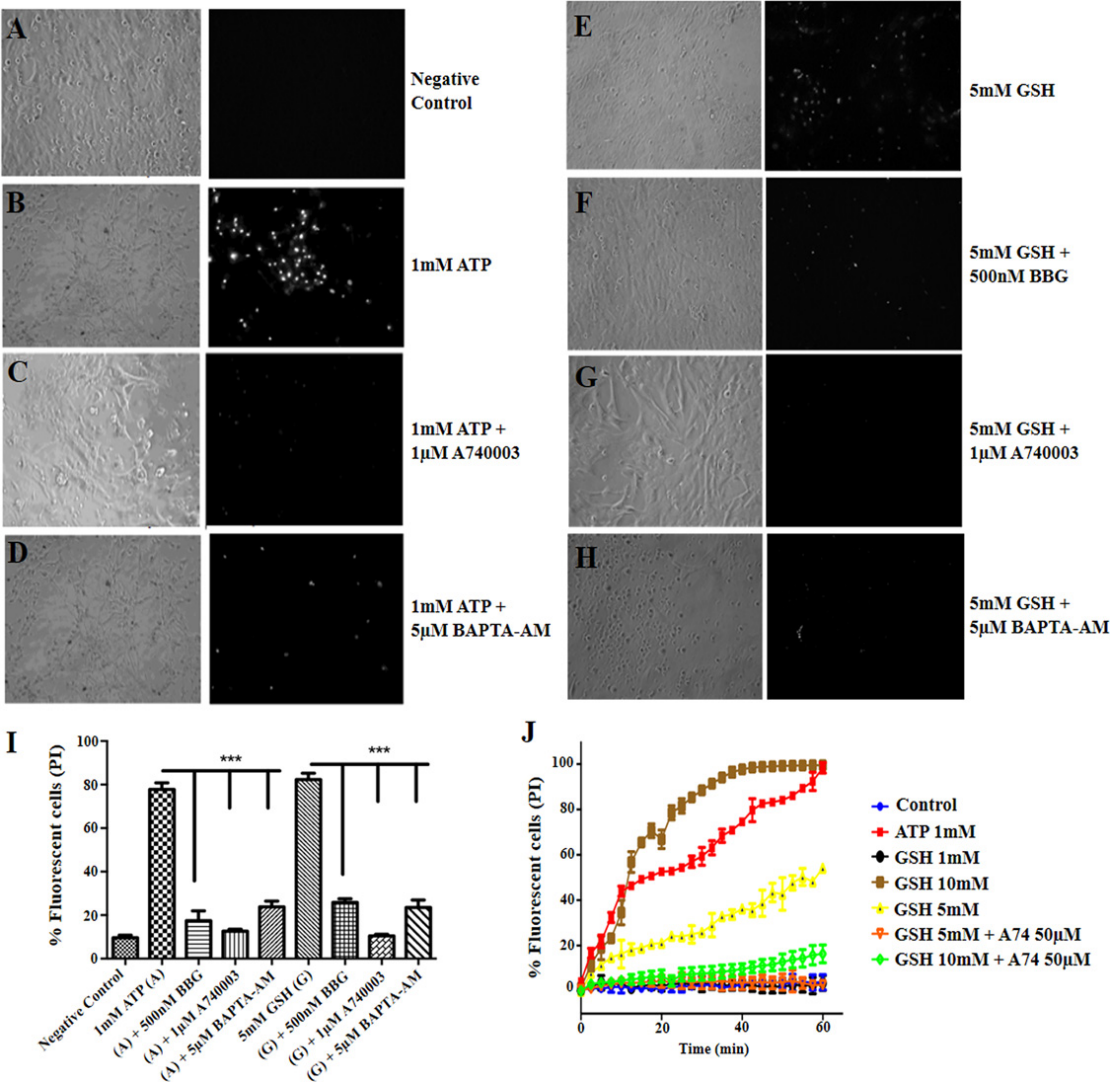
GSH induced PI uptake is inhibited by P2X7 receptor antagonists in Müller glia cell culture. Permeabilization in Müller glia cells depends on P2X7 receptor. (A-H) Left panels are phase contrast, while right are fluorescence images showing PI uptake. No drugs were added (A), or 1 mM ATP (B), and in the presence of 1 μM A74003 (C) or 5 μM BAPTA-AM in avian Muller glia (D). Alternatively, 5mM GSH was added (E), plus 500 nM BBG (F), or with 1 μM A74003 (C) or 5 μM BAPTA-AM (H). P2X7 receptor antagonists or BAPTA-AM were added 10 minutes before ATP or GSH for 20 min. Quantification of the percentage of fluorescente cells is shown in I. N = 3 plates done in triplicate. (J) GSH induced PI uptake is inhibited by P2X7R antagonists. Permeabilization assays with 1 (in black), 5 (in yellow) and 10 mM (in brown) GSH measured in intervals of 2.5 minutes until 60 minutes. A740003 was added for 10 minutes before 5 mM GSH treatment (orange) or 10 mM GSH (in green). Negative control (saline) is in blue while cells stimulated with 1 mM ATP are in red. These signals are representative of duplicate measurements performed in 2–3 independent days.

Avian Müller cells in culture express P2X7 receptors [28] and ATP is known to permeabilize mice cortical astrocytes [22] through a phenomenon described as pore dilation. Propidium iodide (PI, MW 668), is a molecule commonly used to measure cell permeabilization Therefore, we asked if addition of ATP or GSH to avian Müller cells in culture could induce PI uptake in a P2X7 receptor dependent manner. As shown, 9.6% of the Müller cells incubated in saline were able to uptake PI after 30 min (Fig 7A and 7I). Addition of 1mM ATP increased PI uptake to 78% of Müller cells (Fig 7B and 7I). Preincubation of 1μM A74003 (Fig 7C and 7I), a P2X7 antagonist (10 min before ATP) reduced the uptake of PI to 13% of plated glia cells. Also, preincubation of 5μM BAPTA-AM, a cell permeant chelator, reduced the uptake of PI induced by ATP to 24% of the plated cells (Fig 7D and 7I) suggesting the involvement of intracellular calcium in this phenomenon. Alternatively, 5mM GSH added to Müller cells increased PI uptake by 82% of the plated cells (Fig 7E and 7I). Addition of 500 nM BBG 10 min before 5 mM GSH inhibited PI uptake to 26% of the plated glia cells (Fig 7F and 7I). As expected, addition of 1μM A74003 also blocked PI uptake induced by 5 mM GSH to 10% of the Müller cells (Fig 7G and 7I). Finally, preincubation of 5μM BAPTA-AM reduced the uptake of PI induced by GSH to 24% of the plated cells (Fig 7H and 7I). Quantification of the percentage of fluorescent cells permeabilized by ATP or GSH is shown if Fig 7I, while the kinetics of PI uptake is shown time- and concentration-dependent manner (Fig 7J). Altogether our data strength the ideas that GSH interacts with P2X7 receptor inducing pore dilation to uptake PI.

### GSH does not induce cell death in retinal cells in culture

**Fig 8:**
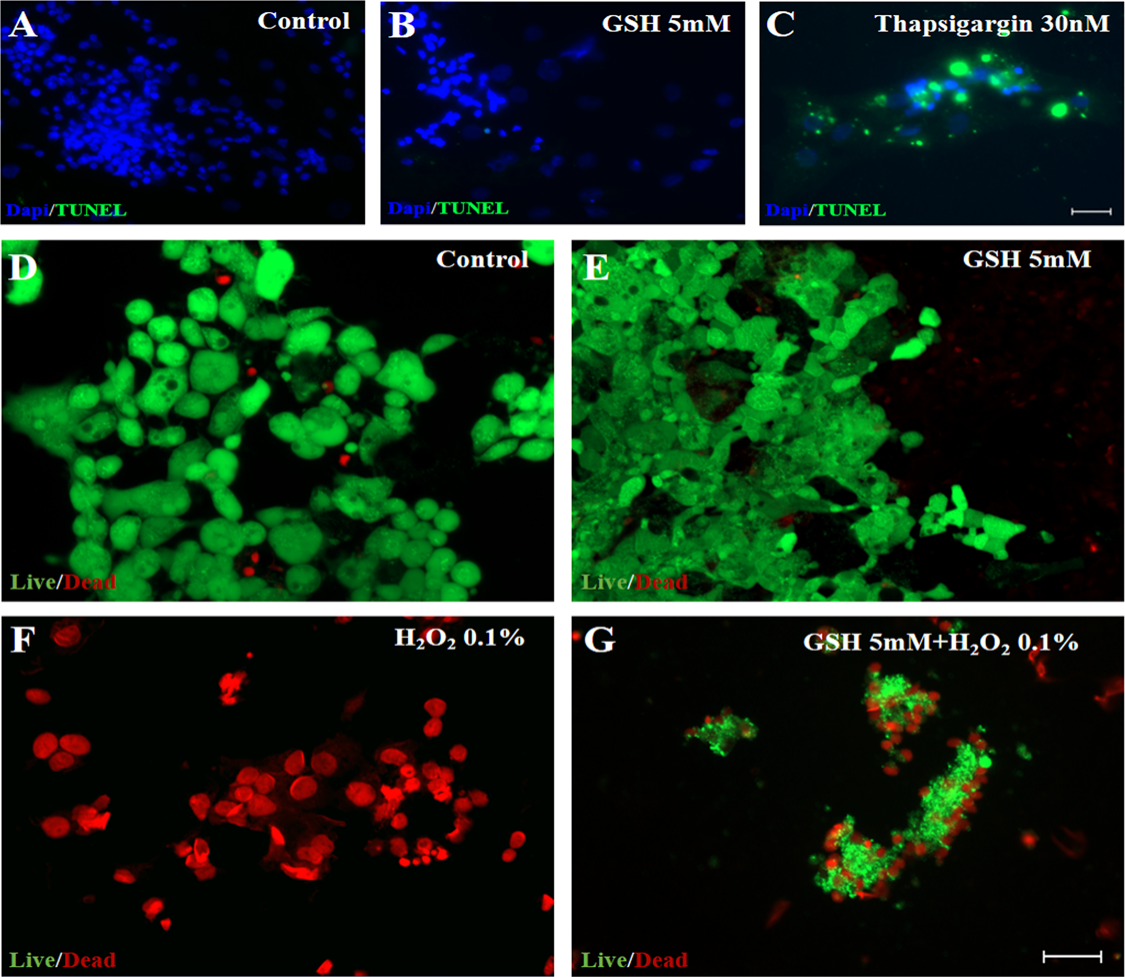
GSH does not induce death in retinal cells in culture. GSH does not induce cell death in Müller glia cells in culture A-C. A 60 seconds 5mM GSH does not induce cell death, but 30nM thapsigargin induce apoptosis. A and B. No TUNEL positive cells are found in the control or in the GSH-treated (5mM) cells in cultures. C. Retinal cells in culture treated with thapsigargin (30nM) show innumerous TUNEL-labeled regions. 50μm scale bar. N = 3, performed in triplicate. D-G. Live and Dead viability assay for a 60s GSH 5mM stimulus. D. Control or E. GSH 5mM treated cultures show few or none labeled cells (red as propidium iodine positive cells), while F. H_2_O_2_ 0.1%. treated cultures were essentially labelled with propidium iodine (red). G. GSH 5mM treated cultures for 60s is sufficient to protect partialy retinal cells in culture. D. 50μm scale bar. Total of three individual experiments (n = 3).

It is known that Ca^+2^ shifts in Müller glia cells may represent part of a protective mechanism, however a sudden intracellular increase of Ca^+2^, frequently leads to apoptosis. In order to rule out this possibility, we checked nuclear integrity using the TUNEL assay to detect DNA fragmentation. As shown, 5 mM GSH does not induce an increase in the number of TUNEL positive cells compared to control culture (Fig 8A and 8B). For positive control of TUNEL, cells were treated with the inhibitor of the endoplasmic reticulum calcium ATPase thapsigargin. Previously, we described that thapsigargin induces apoptosis of photoreceptors in rat retinal tissue [22]. Here, we show that treatment with 30nM thapsigargin, for 24 hours, dramatically increases TUNEL positive cells (Fig 8C) in chicken retinal cell culture. In addition, we also performed the Live and Dead assay to check the possibility of cell death induced by GSH at the millimolar level. We found that 5mM GSH treated cultures show few or none labeled cells for propidium iodine (red), while 0.1% H_2_O_2_ treated cultures had essentially all cells labeled with propidium iodine (Fig 8D and 8E). Finally, GSH 5mM treated cultures for 60s was sufficient to protect partially retinal cells in culture, when previously incubated with 0.1% H_2_O_2_ (Fig 8F and 8G). These data indicate that 5mM GSH does not induce cell death of retinal cells.

### Redox capacity is enhanced following a short pulse of GSH

**Fig 9:**
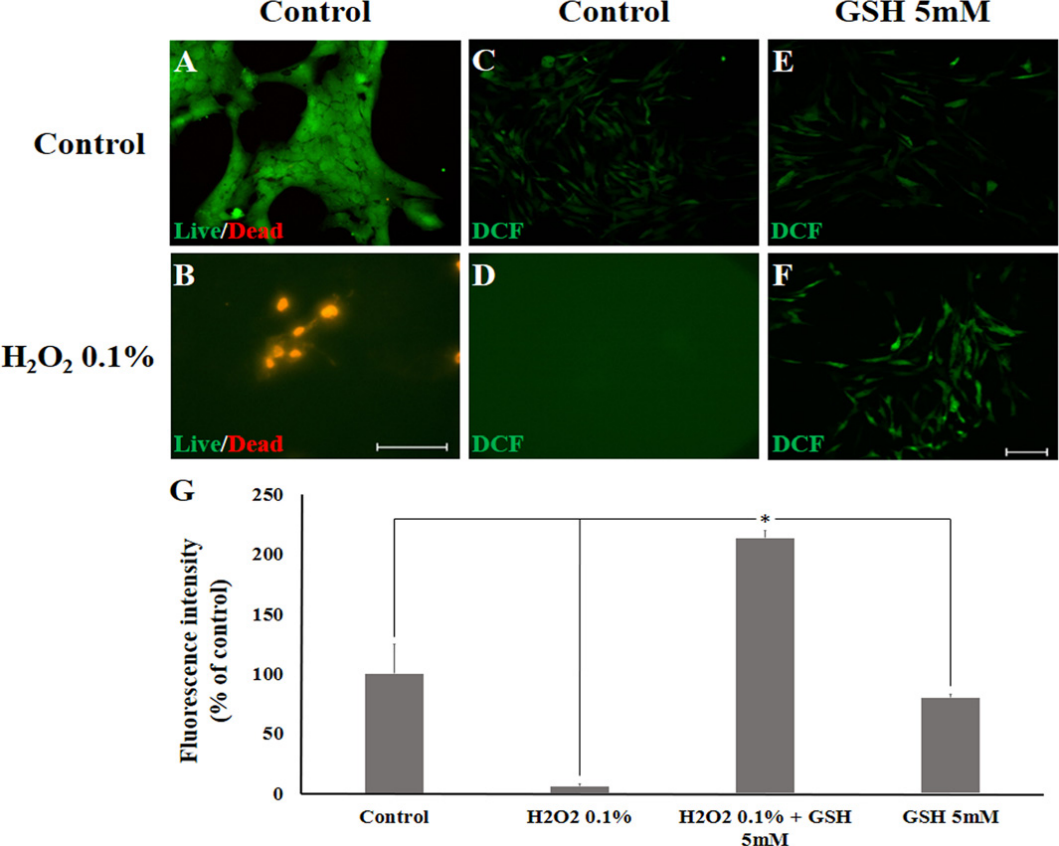
Analysis of ROS activity based on a DCF assay in Müller cells. (A and B.) Live/Dead cell viability in the presence of 0.1% H_2_O_2_ (C to F) ROS activity (DCFH oxidation) in the presence of 5mM GSH or compared to control levels. (C and D.) Cells incubated with hydrogen peroxide (H_2_O_2_ 0.1%) did not survive or were extremely stressed. (E and F.) Cells were able to survive when pre-incubated for 60 seconds with 5mM GSH followed by H_2_O_2_-induced (0.1%) oxidative stress. G. Even through intense ROS activity, cells incubated with H_2_O_2_ 0.1% + GSH 5mM were protected and able to survive in H_2_O_2_-induced toxicity condition. At least five (n = 5) individual experiments were performed for each condition. B and F. 100μm and 50μm scale bars, respectively. *Calculated significance was at least *p* < 0.05.

**Fig 10:**
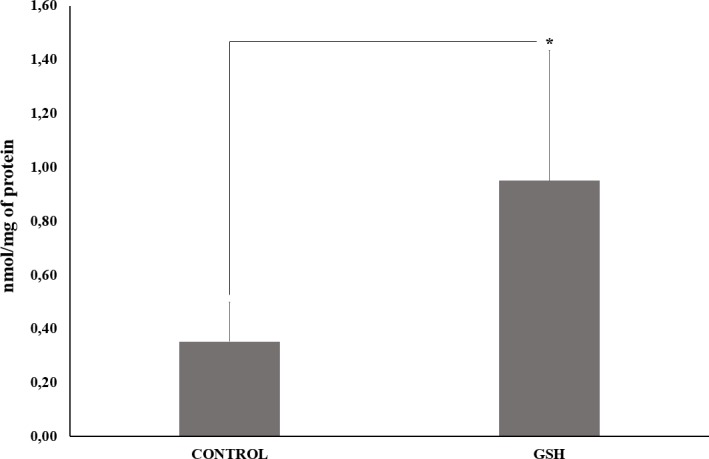
Quantification of GSH in Müller glial cells after a 60 seconds incubation. Intracellular GSH concentrations after a 60s incubation with either Krebs solution or with 5mM GSH. Incubation time aimed to mimic single cell calcium imaging experiments, where cellular response to antioxidants were evaluated (Figs 1–6). *Calculated significance was at least *p* < 0.05.

In order to check whether intracellular redox capacity is altered after a 60 seconds GSH pulse, we tested the effect of this short pulse on the oxidative insult elicited by 0.1% H_2_O_2_ on purified Müller glia for 30 minutes. It has been previously shown that this deleterious oxidative condition renders 100% of death in Müller glia cells in culture [20]. We first confirmed the toxicity induced by this condition on Müller glia (Fig 9A–9D), decreasing cell viability. Then, we found that a short GSH pulse protects Müller glia from oxidative damage after 30 min of incubation with 0.1% H_2_O_2_ as assessed by CM-H2DCFDA oxidation (Fig 9E and 9F). Quantification of fluorescence intensity (% of control) confirmed these results (Fig 9G). It was also observed that intracellular GSH levels in Müller glia pre-incubated for 60 seconds with GSH are increased, as compared to cells incubated only with PBS (Fig 10). Taken together, our data indicate that the ability of these cells to deal with oxidative insults is quickly enhanced by incubation with GSH.

## Discussion

In the last two decades, the concept of gliotransmitters emerged comprising secreted compounds by astrocytes that synchronize networks and modulate neuronal function. The main signaling molecules that fit this definition are glutamate, ATP and D-serine [29]. Nowadays astrocytes are clearly recognized as active elements in the nervous system, and in the vertebrate retina, this compartment is devoted to Müller glia cells [30]. Müller glia-neuronal interactions are observed in every class of the retinal neurons, and the interaction between these cells is mostly bidirectional. Recent evidence points to the critical role of calcium in the release process by glia [31] and all three cited gliotransmitters operate in calcium permeable receptors.

Our data suggest that glutathione could be added to the class of signaling molecules secreted by glial or neuronal cells, and we show that GSH acts solely by controlling glial-based Ca^2+^ shifts. We found that 5mM GSH, but not micromolar concentrations, induced a sharp and fast Ca^2+^ increase, occurring exclusively in Müller glia cells in culture. This effect is completely inhibited in the presence of a P2X7 purinergic receptor inhibitor (BBG), but not in the presence of glutamatergic ionotropic NMDA and non-NMDA antagonists (MK-801 + DNQX). Interestingly, a study using single cell calcium fluorescence has shown that 300 μmol/L GSH raises cytosolic Ca^2+^ in rat hippocampal neurons in culture, while GSSG fails to produce such effect. This effect is mimicked by S-nitrosoglutathione (GSNO) and blocked by Mg^2+^ and AP5, a NMDA receptor inhibitor [32]. In our study, GSH acted selectively on Müller glial cells, and glutamate receptor antagonists did not block this effect (Figs 2 and 4). GSH failed to activate enriched neuronal cells in culture (Fig 3). Our data suggest a weak response by retinal neurons in culture due to probably a later consequence of previous glial activation by millimolar concentrations of GSH (see Fig 2B). The intracellular levels of GSH in the nervous system are around 4mM, and GSH is synthesized in all cells in the brain. GSH is slightly predominant in glia while in neurons show a concentration of 2.5mM [4], [5].

It has been a matter of debate if GSH could serve as a neuromodulator or as a neurotransmitter (reviewed in [2, 8, 9]). Evidence show the presence of high-affinity binding sites for GSH and GSH derivatives in pig cerebral cortical synaptic membranes [10]. In addition, thiol-containing compounds, may participate in reduction–oxidation (redox) reactions to alter the biophysical properties of various ionotropic receptors and ion channels. Indeed, several findings support the fact that GSH and its derivatives have some affinity for the NMDA recognition domain in a manner independent of the thiol moiety [9]. It was also shown that GSH releases [^3^H] dopamine in mouse striatal slices [8]. In addition, astrocytes have the ability to secrete GSH into the extracellular space [11], [12]. This secretion of GSH serves as a precursor supplier for other brain cells.

In an attempt to clarify the mechanism through which GSH induces calcium shifts in Müller glia, we found that the ability of these cells to deal with oxidative insults is quickly improved by incubation with GSH. In this scenario, disruption of cellular redox capacity, which depends greatly on GSH levels, elicits oxidative stress and also nitrosative stress in neural cells [33],[34]. Since under physiological conditions the reduced GSH is the major form ranging from 10 to 100-fold higher concentrations when compared to glutathione oxidized species [35], it is unlikely that GSH effects on calcium shifts are related to redox environment. However, it cannot be ruled out that the effects of GSH inducing calcium shifts in Müller glia are, at least partially, secondary to intracellular redox modifications instead of extracellular signaling.

Our data indicate that GSH induces a dose-dependent release of GABA in the avian retinal tissue. We used different types of cell cultures to show that Müller cells, but not isolated neuronal cells, were activated by GSH, inducing Ca^2+^ increase. Also, [^3^H] GABA release induced by GSH was shown in cultured Müller cells or in dense mixed neuron-glia cultures. Mixed retinal culture system, and the retinal tissue used in the present work are composed of neurons and Müller cells, and both cell types contain GABA. In particular, a subset of amacrine neurons uses GABA as a neurotransmitter [36]. Both amacrine neurons and Müller cells are able to release GABA in retinal tissue. This phenomenon have been described as Ca^2+^-dependent and independent as well as Na^+^-dependent and independent mechanisms [21, 37]. It is well documented that all these mechanisms of GABA release can be regulated by different neurotransmitters such as glutamate, D-aspartate and nitric oxide [21, 38, 39].

In the present study, we showed that GSH evokes GABA release from retinal cells. However, we could not determine precisely which retinal cell types were the source of GABA released during GSH treatment. Based in previous studies and some results of our study, we hypothesized that both neurons and Müller cells could be involved in GABA release induced by GSH. Previous work from our laboratory have shown that depolarizing agents (KCl or veratridine) or transmitters (glutamate or GABA) are not able to release [^3^H] GABA in purified avian Müller glia cells in culture [16]. Now we show that [^3^H] GABA can be released by GSH by these cells (Fig 6). In addition, it is possible that calcium increase induced by GSH in Müller cells might release a mediator that could potentially induce GABA release from amacrine neurons in the mixed culture, either by exocytotic mechanism (Ca^2+^-dependent) or by reversal of the carrier (Na^+^-dependent) activity. However, we cannot discard a possible GABA release mediated by Ca^2+^-independent mechanism present in both retinal cells that produce/accumulate GABA. In fact, it is well documented that more than 80% of GABA released from chick retinal cell after GAT-1/3-like transporter mediates glutamatergic activation [21]. In addition, GSH treatment evokes significant alterations in GAT-1 activity, since it is documented that alteration in the redox status of the transporter alters its activity [40]. Indeed, recent data from our lab indicate that GABA uptake by GAT-3 transporter is influenced by glutamate in cultured Müller cells [20]. P2X7 seems to be an essential target for the role of GSH in Müller glia cells; we are still investigating a role of this receptor in terms of GABA release or neuroprotection in terms of GSH signaling. Since ATP induces cell death in mixed neuronal-glial cell cultures [28], but not in purified cultures of neurons or glia [28], the effect of GSH and GABA could strengthen neuroprotection in the retinal tissue and Müller cells could again exert a central role in this matter.

ATP is the classical ligand for P2X7 receptors. However, our data suggest that GSH might also be operating directly at the P2X7 receptor, as the phenomenon is blocked by BBG or A74003, and also by addition of BAPTA-AM, which blocked the PI uptake in glia cells. In this scenario, other ligands to P2X7 receptors have already been suggested in the literature. A paper by [41] indicates that the low MW nicotinamide adenine dinucleotide (NAD) interacts at the P2X7 receptor in human monocytes, opening the intrinsic ion channel attached to the receptor followed by recruitment of a hemi channel protein involved as part of the pore, pannexin-1 ([42]). The authors showed that the activation of P2X7 receptor induces the release of interleukin-1β (IL-1β) through a covalent bond with ART.2 in lymphocytes, and concluded that signaling via NADPH oxidase activity is mandatory for the release of mature IL-1β induced by P2X7 receptors stimulation. It is uncertain for us whether such pathway could be operating in glia cells, but it cannot be ruled out that GSH might have a role in this redox activity. Furthermore, Molz and colleagues [43] demonstrated that preincubation with docosahexaenoic acid, an antioxidant long-chain n-3 polyunsaturated fatty acid, protects cellular viability in mice hippocampal slices following oxygen and glucose deprivation, and that the neuroprotection afforded was not dependent on glutamate transport, but rather it involved the modulation of P2X and A1/A2B adenosine receptors. The authors suggested a direct interaction between the antioxidant compound docosahexaenoic acid and purinergic receptors, including P2X7 receptor, similarly to what we are currently proposing for GSH in our present work.

In conclusion, GSH may act as a signaling molecule in the neuronal-glial circuit, with relevant role in protection from excitotoxic insults. Neuroprotection would be potentiated by GABA release induced by GSH. Our work proposes that Müller cells sense GSH as a signal to activate P2X7 receptors leading to calcium increase. Both amacrine neurons and Müller cells are able to release GABA in retinal tissue. In this sense, in addition to the well-known GSH antioxidative effects, P2X7 purinergic receptors could mediate a calcium-induced part of a neuro/protective mechanism. It is well demonstrated that increased levels of glutamate in the synaptic cleft induces oxidative stress and excitotoxicity in neuronal cells from brain and retina. In addition, it is also known that hyperpolarization GABA-induced protects neurons against toxicity induced by glutamate. We have previously demonstrated that glutamate induces GSH release from retinal cells; but until the present study the role of GSH as a regulatory molecule of GABA release was not described. Our hypothesis is that the excess of glutamate induces GSH release, which evokes GABA release mediated by P2X7 receptors protecting neuronal cells against oxidative stress and toxicity induced by glutamate.
